# β-Amyloid: the key peptide in the pathogenesis of Alzheimer’s disease

**DOI:** 10.3389/fphar.2015.00221

**Published:** 2015-09-30

**Authors:** Xiaojuan Sun, Wei-Dong Chen, Yan-Dong Wang

**Affiliations:** ^1^Key Laboratory of Receptors-Mediated Gene Regulation and Drug Discovery, School of Medicine, Henan UniversityKaifeng, China; ^2^State Key Laboratory of Chemical Resource Engineering, College of Life Science and Technology, Beijing University of Chemical TechnologyBeijing, China

**Keywords:** amyloid β peptide, Alzheimer’s disease, amyloid precursor protein, biogenesis, animal models

## Abstract

The amyloid β peptide (Aβ) is a critical initiator that triggers the progression of Alzheimer’s Disease (AD) via accumulation and aggregation, of which the process may be caused by Aβ overproduction or perturbation clearance. Aβ is generated from amyloid precursor protein through sequential cleavage of β- and γ-secretases while Aβ removal is dependent on the proteolysis and lysosome degradation system. Here, we overviewed the biogenesis and toxicity of Aβ as well as the regulation of Aβ production and clearance. Moreover, we also summarized the animal models correlated with Aβ that are essential in AD research. In addition, we discussed current immunotherapeutic approaches targeting Aβ to give some clues for exploring the more potentially efficient drugs for treatment of AD.

## Introduction

Alzheimer’s disease (AD), also known as Senile Dementia, is a most common age-related neurodegenerative disorder. More than 11 million people per year are estimated to suffer from this disease by 2050, leading to higher cost as well as more burdens on public health and society (Alzheimer’s, 2014, 2015). Featured by progressive memory loss and cognitive dysfunction, AD induces the loss of motor functions and personality changes, and eventually leads to death. Histopathologically, AD is mainly characterized by extracellular senile plaques (SPs) and intracellular neurofibrillary tangles (NFTs), which results in the loss of neurons and synapses and finally causes gross atrophy of the brain. NFTs are formed by the regulation of the abnormally hyperphosphorylated and glycosylated microtubule-related tau protein, whereas SPs are associated with the aggregation and deposition of amyloid β peptides (Aβ) (Mattson, 2004).

Aβ accumulation is considered to be the distinct morphological hallmark of early onset of AD and it is also proposed to be an activator to induce the sequential lesion events induced by the aggregation of P-Tau. Therefore, Aβ is predicted to be the most potentially efficient target of the drug therapies (Karran et al., 2011). Here, this review will focus on this peptide with the aspects of its biogenesis, regulations, as well as degradation and clearance to elucidate the potential significance of these processes for the clinic treatment of AD.

## The A**β** Biogenesis, Toxicity, Production, and Clearance

### The Biogenesis of A**β**

**FIGURE 1:**
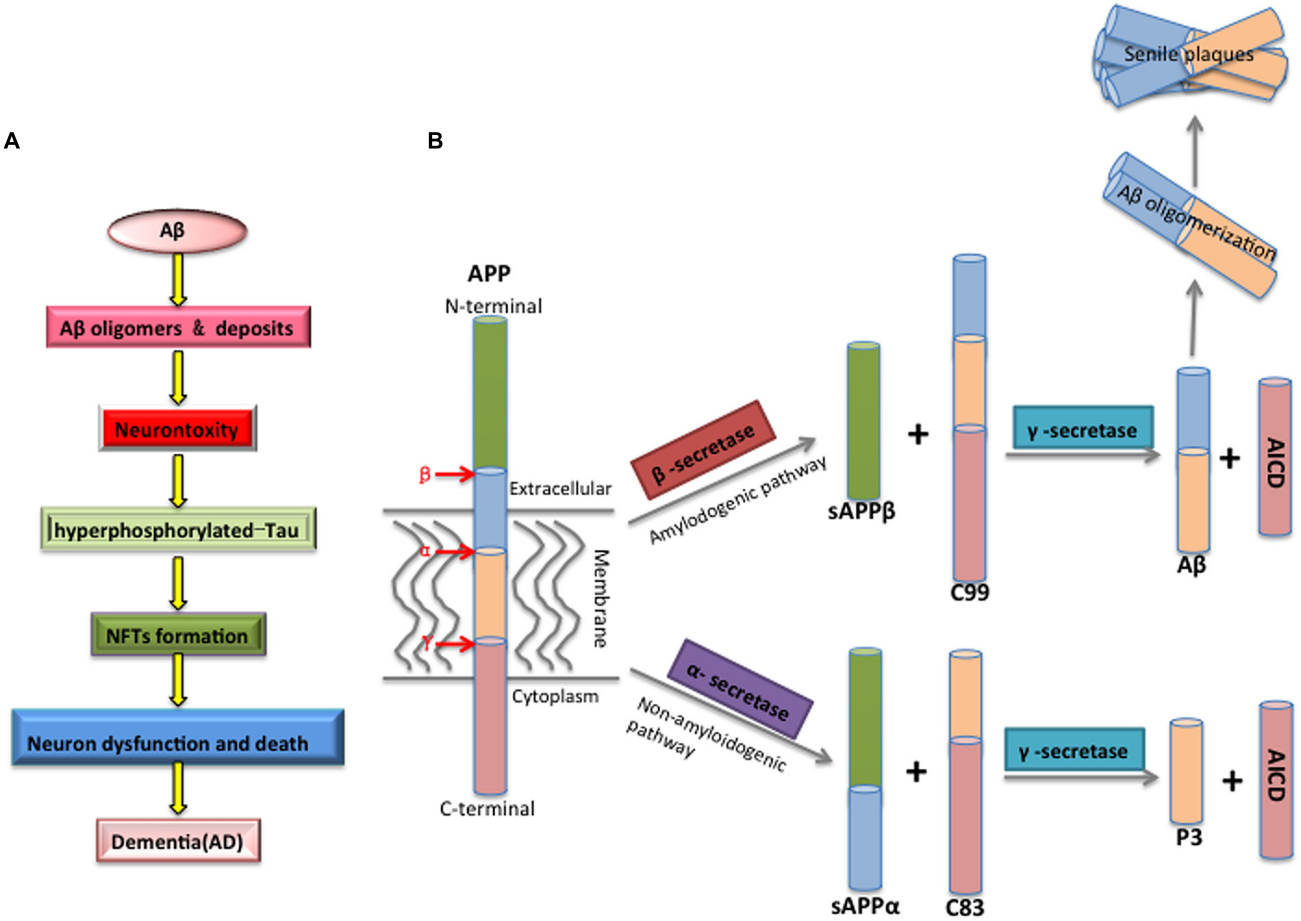
**(A)** The mechanism of Aβ toxicity. Accumulating Aβ will initially results in Aβ oligomerization, gradually deposits as the forms of fibrils and senile plaques. Furthermore, Aβ aggregation alters the kinase/phosphatase activity that leads to the Tau protein hyperphosphorylated, which causes the formation of neurofibrillary tangles (NFTs), and eventual synaptic and neuronal dysfunction and AD. **(B)** The proteolytic processing of the amyloid precursor protein (APP) and Aβ biogenesis. APP is a transmembrane glycoprotein with a large luminal domain and a short cytoplasmic domain, and it is processed through amyloidogenic or non-amyloidogenic pathway. The amyloidogenic pathway is the process of Aβ biogenesis: APP is firstly cleaved by β-secretase, producing soluble β-APP fragments (sAPPβ) and C-terminal β fragment (CTFβ, C99), and C99 is further cleaved by γ-secretase, generating APP intracellular domain (AICD) and Aβ. The non-amyloidogenic pathway is an innate way to prevent the generation of Aβ, as APP is firstly recognized by α-secretase within Aβ domain, generating soluble α-APP fragments (sAPPα) and C-terminal fragment α (CTFα, C83), C83 is then cleaved by γ-secretase, producing non-toxic P3 and AICD fragments.

The factors involved in the pathogenesis of AD have been intensely investigated, however, the mechanisms governing this disease are not fully understood and remain debated. One prevailing proposal is the amyloid cascade hypothesis positing Aβ as the initiator of subsequent events that leads to AD (**Figure 1A**) (Hardy and Selkoe, 2002): Aβ peptides spontaneously aggregate and deposit into soluble oligomers, fibrils and SPs, which then induces oxidative injury, microglial and astrocytic activity as well as alters kinase/phosphatase activity, eventually leading to the neuronal death. Howerver, whether Aβ acts on tau aggregation is still debated (Musiek and Holtzman, 2015).

Aβ is a small protein composed of 39–43 amino acids with a variety of biophysical states. There are two major isoforms of Aβ, soluble Aβ_40_, and insoluble Aβ_42_, and the latter peptide showing higher percentage concentration in AD patients is more prone to aggregate (Burdick et al., 1992; Gravina et al., 1995; Kim et al., 2007). In a physiological condition, more than 90% of Aβ is in the form of Aβ_40_ while less than 5% is generated as the longer form of Aβ_42_.

Aβ is derived by proteolysis of an evolutionary conserved large transmembrane amyloid precursor protein (APP) through cleavage of β-secretase followed by γ-secretase. Mutations in the gene encoding APP are the main causes of familial AD (FAD; Chartier-Harlin et al., 1991; Goate et al., 1991). APP can also be processed by α-secretase via non-amyloidogenic pathways to produce non-toxic fragments, which is thought to antagonize Aβ generation (**Figure 1B**; Gandy et al., 1994; Sahlin et al., 2007).

Most of intracellular Aβ normally distribute in the neuronal cytosol, but it is also colocalized with different organelles dependent on where APP, β- and γ-secretase reside. In particular, it has been reported to be produced in the secretory pathway related organelles including endoplasmic reticulum (ER), medial Golgi saccules as well as trans-Golgi network (Hartmann et al., 1997; Greenfield et al., 1999). It has also been found to be correlated with the endocytic endosomes/lysosomes and autophagic vacuoles (Koo and Squazzo, 1994; Yu et al., 2004). Besides, Aβ also resides in mitochondria (Muirhead et al., 2010).

### Toxicity of A**β**

The physiological role of Aβ is still unknown, but it indeed exists throughout life in healthy individuals. One possible function is to inhibit the activity of γ-secretase in a negative feedback way (Kamenetz et al., 2003).

Aβ aggregation is considered to be the primary reason for the neurotoxicity in the classic view, and Aβ oligomers are the most neurotoxic form (Walsh et al., 2002). Three “developed-stimulators” may facilitate the aggregation process. The absolute levels of Aβ_42_ increased by production via APP processing, the ratio of Aβ_42_ to Aβ_40_ elevated due to the decreased level of Aβ_40_ or the soluble oligomeric Aβ (Glabe, 2005; Walsh and Selkoe, 2007). These potential stimulators further promote the accumulation and deposition of Aβ to develop into SPs, which eventually contributes to AD pathology. Moreover, Aβ-induced apoptosis through interaction with cell surface receptors and proteins is also thought to dedicate to the dysfunction of neuronal system (Small et al., 2001; Zhu et al., 2015).

The aggregation of Aβ might also promote free radicals such as reactive oxidative species (ROS) to react rapidly with several moieties of proteins and lipids, whose structures or functions are then altered to potential “toxic” oxidized proteins and peroxided lipids. Protein oxidation may cause harm to the membrane integrity or damage the sensitivity to oxidative modification of the enzymes such as glutamine synthetase (GS) and creatine kinase (CK), which are critical to neuronal function (Aksenov et al., 1995; Yatin et al., 1999). Lipids peroxidation usually causes the toxic product such as 4-hydroxy-2-nonenal (HNE) and 2-propenal (acrolein) that migrates to different parts of the neurons to cause multiple deleterious alterations of cellular function. It includes loss of Ca^2+^ homoeostasis, inhibition of ion-motive ATPases and glial cell Na^+^-dependent glutamate and disruption of signaling pathways, all of which are associated with neuronal death (Mark et al., 1995; Varadarajan et al., 2000; Ezeani and Omabe, 2015). Aβ-induced oxidative stress has also been reported to cause the DNA oxidation, leading to DNA damage (Varadarajan et al., 2000).

Continuous Aβ aggregation or sustained elevation of Aβ would cause a chronic response of the innate immune system by activating microglia through some immunological receptors such as Toll-like Receptors 2 (TLR2), TLR4, TLR6, their co-receptors CD14, CD36, and CD47, which can probably destroy functional neurons by direct phagocytosis (Weggen et al., 2001; Neniskyte et al., 2011; Liu et al., 2012). Besides, it also results in inflammatory response, concomitantly releasing a lot of inflammation related mediators including complement factors, eicosanoids, chemokines, and proinflammatory cytokines, which can impair microglial clearance of Aβ and the neuronal debris and increase microglia-mediated neuronal death and loss of neuronal synapses, contributing greatly to AD pathogenesis. Aβ deposition also induces tau pathology by promoting the intraneuronal formation of NFTs which consist of hyperphosphorylated tau proteins. It influences the late-stage of AD pathogenesis. The process is probably mediated by the microglia-driven neuroinflammatory response or by indirectly regulating kinase/phosphatase activity (Heneka et al., 2015).

In addition, Aβ precursor APP accumulation at mitochondria membrane can cause mitochondrial dysfunction by blocking the translocation of other mitochondrial inner molecules/proteins and disrupting the electron-transport chain (ETC; Anandatheerthavarada et al., 2003; Devi et al., 2006), which may in turn increase excessive Aβ generation to result in more toxicity. Excessive Aβ can also increase mitochondrial ROS production to induce mitochondrial fragmentation by activating mitochondrial fission proteins Drp1 and Fis1 (Barsoum et al., 2006). Aβ localized in mitochondria can bind to two pro-apoptotic factors including Aβ-binding alcohol dehydrogenase (ABAD) and cyclophin D (CypD), consequently increasing neurodegenerative cell death that may be toxic to neurons (Lustbader et al., 2004; de Moura et al., 2010). Hence, there may be a vicious feedback loop between increased Aβ production and mitochondria dysfunction.

### Regulations of A**β** Production and Clearance

Because of the key role of Aβ in AD pathogenesis, it has been well accepted that reducing Aβ production or enhancing Aβ clearance may be a putative way to inhibit the cascade of Aβ-induced pathological events.

Aβ biogenesis is tightly correlated with APP metabolism, including processing and trafficking. There are three isoforms of APP, APP695, APP751, and APP770 (Goate et al., 1991). APP695 lacking the Kunitz-type protease inhibitor (KPI) domain is predominantly expressed in neurons while the other two isoforms are distributed in most tissues (Kang and Muller-Hill, 1990; Rohan de Silva et al., 1997). Some evidence show that APP751 and APP770 up-expression in brains are primarily associated to Aβ deposition (Menendez-Gonzalez et al., 2005; Bordji et al., 2010). APP processing is mainly regulated by α, β, and γ-secretases (**Table 1A**). Alpha-secretase plays an essential role in precluding the generation of intact Aβ on account of the cleavage site within the Aβ domain. As a membrane-bound endoprotease, α-secretase usually cleaves APP at plasma membrane (Sisodia, 1992). Several members of the a Disintegrin and metalloproteinase (ADAM) family listed in **Table 1A** have been reported to possess α-secretase activity, which is responsible for APP processing (Koike et al., 1999; Harold et al., 2007; Tanabe et al., 2007; Kim et al., 2009). β- and γ-secretases are devoted to Aβ production via amylodogenic pathway (**Figure 1B**). Beta-site APP cleaving enzyme 1 (BACE1) and BACE2 are the β-secretases while γ-secretase is complex and composed of presenilins (PSEN1 or PSEN2), Nicastrin, Presenilin enhancer 2 (PEN2), and anterior pharynx defective 1 (APH-1; De Strooper, 2003). Substantial evidence has shown that manipulation of these secretases could perturb the generation of Aβ. For example, with the α-cleavage abolished in ADAM17-deficient cells affectd Aβ generation (Buxbaum et al., 1998). Knock-out of BACE1 in mice completely depleted neuronal Aβ secretion (Cai et al., 2001). Mutations of PSEN1 and PSEN2 affected APP cleavage, thereby altering Aβ production (Wang et al., 2010). Moreover, regulators related with these secratase, such as the γ-secretase activating protein (GSAP) and CD147, are also likely to be involved in the generation of Aβ (Zhou et al., 2005; He et al., 2010).

**Table 1A:** Member proteins of three secretases.

Secretase	Members in mammals
α-secretase	ADAM9, ADAM10, ADAM12, ADAM17, ADAM19, and MDC9
β-secretase	BACE1 and BACE2
γ-secretase	PSEN1, PSEN2, nicastrin, APH-1, and PEN-2

Like other type I transmembrane proteins, APP is synthesized and translocated into ER followed by matured in the Golgi apparatus where APP is mainly concentrated in neurons at the steady state (Hartmann et al., 1997; Xu et al., 1997; Greenfield et al., 1999; Caster and Kahn, 2013). And then APP would traffic through the constitutive secretory pathway. Once reaching the cell surface, it is either cleaved by α-secretase to produce sAPPα (Sisodia, 1992) or rapidly re-internalized by recognition of a “YENPTY” motif and subsequently recycled back to the cell surface by the recycling compartments or delivered to the lysosome for degradation through the endosomal–lysosomal systems (Golde et al., 1992; Caster and Kahn, 2013). Generally, promoting APP delivery or inhibiting APP internalization from the cell surface favors the non-amyloidogenic processing, thereafter antagonizing the generation of Aβ. Elevating retention of APP in acidic compartments such as endosomes greatly adds the chances for amyloidogenic processing and consequent Aβ production. Mutations within the “YENPTY” internalization motif have been addressed to block APP internalization and consequently decrease Aβ generation (Perez et al., 1999). In contrast, mutation within extracellular KPI domain existing in APP751 and APP770 that assists APP sorting to plasma membrane causes APP retention in the ER, thereby elevating the Aβ production (Ben Khalifa et al., 2012). Synaptic transmission indicated to accelerate APP endocytosis has also been shown to result in increasing the level of secreted Aβ (Cirrito et al., 2005). Some general modulators that could regulate APP trafficking, such as dynamin I (Carey et al., 2005), the RAB GTPase family including RAB1B, RAB6, RAB8, and RAB11 (Huber et al., 1993; Dugan et al., 1995; McConlogue et al., 1996; Thyrock et al., 2013; Udayar et al., 2013), and the SNX family including SNX17 and SNX33 (Lee et al., 2008; Schobel et al., 2008), have also been found to be associated with Aβ generation. In addition, factors that function in the trafficking of the three secretases may also change the production of Aβ by affecting APP processing (Wahle et al., 2006; Wen et al., 2011; Bhalla et al., 2012). The G-protein-coupled receptor protein GPR3, which is responsible for the cell surface localization of matured γ-secretase, stimulates Aβ production when it is overexpression (Thathiah et al., 2009).

Proteolytic degradation is thought to take a large part of responsibility in preventing Aβ aggregation or deposition into plaques. The enzymes or proteases in proteolytic degradation play important roles by cleaving Aβ into shorter soluble fragments without neurotoxic effect. The proteases including cathepsin B (CatB), cathepsin D (CatD), Gelatinase A, serine protease factor Xia (FXIa), matrix metalloprotein-9 (MMP-9), neprilysin (NEP), presequence protease (Prep) and the α_2_M complex are involved in Aβ clearance (Saporito-Irwin and Van Nostrand, 1995; Yamada et al., 1995; Hamazaki, 1996; Carvalho et al., 1997; Iwata et al., 2001; Mueller-Steiner et al., 2006; King et al., 2014), while enzymes such as angiotensin-converting enzyme (ACE), endothelin-converting enzyme (ECE), insulin-degrading enzyme (IDE), and uPT and tPA have been found to be involved in the degradation of Aβ (**Table 1B**; Ledesma et al., 2000; Tucker et al., 2002; Eckman et al., 2003; Farris et al., 2003; Hemming and Selkoe, 2005; Baranello et al., 2015).

**Table 1B:** Proteases/enzymes involved in the cleavage of Aβ peptide.

Protease/enzyme	Description
ACE	Angiotensin-converting enzyme
CatB	Cathepsin B, a cysteine protease in lysosome
CatD	Cathepsin D, a cysteine protease in lysosome
ECE	Endothelin-converting enzyme
FXIa	Serine protease factor XIa
Gelatinase A	Secreted endopeptidase
IDE	Insulin-degrading enzyme
MMP-9	Matrix metalloproteinase
NEP	Neprilysin, neutral endopeptidases
PreP	Presequence protease
The plasmin system	Components including plasmin and urokinase-type plasminogen activator (uPA), tissue plasminogen activator (tPA)
α_2_M complex	Serine protease-α_2_ macroglobulin complex

Besides the proteolysis for Aβ degradation, receptor-mediated endocytosis of Aβ that delivers Aβ to lysosome for degradation also contributes to the clearance of toxic Aβ peptide and Aβ deposits. Low-density lipoprotein receptor-related protein 1(LRP1) is considered to be the vital modulator in this process by probably direct binding to Aβ for uptake (Li et al., 2000) or through Aβ receptor such as heparin sulphate proteoglycan (HSPG; Kanekiyo et al., 2011) and GPI-anchored cellular prion protein (PrP^c^; Taylor and Hooper, 2007) to facilitate Aβ trafficking. In addition, Aβ aggregates may also undergo maropinocytosis or phagocytosis for clearance, of which the critical step about actin polymerization is regulated by LRP1(Kanekiyo and Bu, 2014). Apolipoprotein E (ApoE), as a major ligand for LRP1 and an important partner of Aβ, plays dual roles in Aβ clearance (Li et al., 2012; Kanekiyo and Bu, 2014). Moreover, induction of another degrading pathway of autophagy serves to accelerate the clearance of both soluble Aβ and Aβ aggregates (Nixon, 2007).

## Animal Models Related with A**β** for AD

Various types of animal models related to Aβ have been created to dissect the mechanisms for the development and progression of AD, the majority are overexpression transgenic lines (see the summary in **Table 1C**; Oakley et al., 2006; Elder et al., 2010; Do Carmo and Cuello, 2013; Lublin and Link, 2013; Lim et al., 2016).

**Table 1C:** Summary for Aβ related transgenic animal models.

Organism	Aβ related transgenic strains	Description
*Caenorhabditis elegans*	*P_unc-54_::SP:: Aβ^1-42^*	β-amyloid constitutive formation in muscles
	*P_myo-3_::SP:: Aβ^1-42^::long 3′UTR*	Inducible larval paralysis in muscles
	*P_snb-1_::SP:: Aβ^1-42^::long 3′UTR*	Inducible β-amyloid expression in Pan-neurons
	*P_eat-4_::SP:: Aβ^1-42^*	β-amyloid formation in glutamatergic neurons
*Drosophila*	*gmr-Gal4 > UAS-BACE;UAS-dPsn;UAS-APP*	Aβ generated from APP that are cleaved by β-secretase and γ–secretase in retina
	*elav-Gal4 > UAS-Aβ_42_*	Inducible Aβ_42_ expression in the brains
	*gmr-Gal4 > UAS-Aβ_42_*	Inducible Aβ_42_ expression in the retina
	*act5c-Gal4 > UAS-Aβ_42_*	Inducible Aβ_42_ ubiquitous expression
Mouse	Tg2576	Aβ plaques as well as some vascular amyloid are induced by overexpression a mutant form of APP (APPK670/671L)
	APP23	Excessive Aβ production induced by overexpression of mutant human APP carrying the Swedish mutation.
	PDAPP	Expression of mutant human APP carrying the Swedish mutation under the PDGF promoter.
	TgCRND8	Expression of human APP carrying the Swedish and Indiana mutations under the PrP promoter.
	PS1M146V	Expression of human PS1(M146V) under the PDGF promoter.
	APP/PS1	Excessive Aβ production induced by Overexpression of two mutant forms of APPSWE and PSEN1d E9
	5xFAD	Double transgenic APP/PS1 mouse model with co-expression five AD mutations including APP Swedish,Florida and London mutations and PS1 M146L and L286V mutations.
Rat	TgAPPswe	Expression of hAPP751with the Swedish mutation driven by the PDGF promoter
	UKUR28	Expression of hAPP751with the Swedish and Indiana mutations driven by the PDGF promoter
	UKUR25	Expression of hAPP751with the Swedish and Indiana mutations as well as PS1(M146L) driven by the PDGF promoter
	hAPP695	Expression of hAPP695 (wild type) driven by the UbiquitinC promoter
	Tg6590	Expression of hAPP695 with the Swedish mutation driven by the UbiquitinC promoter
	APP21APP31	Expression of hAPP695 with the Swedish and Indiana mutations driven by the UbiquitinC promoter
	PSAPP (Tg478/Tg1116/Tg11587)	Triple transgenic strain carrying expression of hAPP695 with the Swedish mutation under the Rat synapsin I promoter, hAPP695 with the Swedish and London mutations under the PDGFβ promoter and expression of human PS1(M146V) driven by the Rat synapsin I promoter.
	TgF344-AD	Expression of hAPP695 with the Swedish mutation and PSΔE9 under the murine PrP promoter.
	McGill-R-Thy1-APP	Aβ accumulation induced by expression the human APP carrying both the Swedish and Indiana mutation under the control of the murine Thy 1.2 promoter.

Despite the existing innate disadvantages. e.g., the transgenic flies that express both human APP and β-secretase BACE1 displayed Aβ accumulation, the animal models are useful to screen genes involved in APP processing (Ye and Fortini, 1999; Greeve et al., 2004), making a great contribution to the development of this field. The secreted-Aβ model in *Drosophila* is a direct approach to investigate the toxicity caused by Aβ (Finelli et al., 2004; Crowther et al., 2005; Iijima et al., 2008). The *Caenorhabditis elegans* Aβ-expressing models developed in different tissues are also helpful for examining genes involved in Aβ-induced toxicity (Link, 2006; Wu et al., 2006). Phenotypes were also analyzed in zebrafish through high-throughout screen by treatment with Alzheimer’s γ-secretase inhibitors to determine efficient compounds for blocking Aβ generation (Arslanova et al., 2010).

Aβ infusion models are that different species of Aβ peptides is directly injected in the rodent brains. They could mimic the most aspects of AD and deliver experimental results for analysis in a relatively short time (Nag et al., 1999; Harkany et al., 2001; Nakamura et al., 2001). However, these approaches usually induce much higher levels of Aβ in the brains than that exists in the patients, and the results usually vary due to differences in methodology and the concentration of Aβ and the duration treatment. Although most of the models do not show Tau pathology and other shedding fragments from APP processing may also influence neuron systems, transgenic rodent models with overexpression of wild type or mutated human APP can recapitulate some features of AD pathology and provide great convenience to discover more regulators involved in the onset of AD (Clarke et al., 2007; Agca et al., 2008; Leon et al., 2010; Rosen et al., 2012). Nevertheless, no model system is impeccable, further understanding of the molecular mechanisms for Aβ-initiated AD pathology would still be desirable.

## Overviews of Current Therapeutics Targeting A**β**

According to the conventional approaches targeting Aβ, therapeutic strategies focus on reducing Aβ production via inhibition of β- and γ-secretases to prevent Aβ aggregation and facilitate Aβ clearance. However, the results are not so inspiring, as all the strategies have failed in clinical trials. Recently, immunotherapies by two monoclonal antibodies against Aβ have been tried. One is Bapineuzumab that could recognize both soluble and insoluble forms of Aβ; the other one is Solanezumab that targets Aβ central domain and recognizes only soluble Aβ. Yet both of them failed to improve the clinical outcomes in patients in phage III trials (Doody et al., 2014a,b; Salloway et al., 2014), which suggests that targeting Aβ alone might not be enough to impede AD progression and multiple steps of Aβ modulations should be taken into consideration according to the different clinical phenotypes in AD patients. e.g., the activity of Foxp3+ regulatory T cells (Tregs) has been reported to be related with Aβ plaque clearance, suggesting novel immunosuppression curing way (Baruch et al., 2015). Moreover, other approaches besides immunotherapy also need to be explored in order to understand multiple regulations of Aβ for the development of therapies for treating AD.

## Conclusion and Perspectives

The vital role of Aβ as an initiator in the pathology of AD has been well accepted. Aβ production mainly depends on APP processing, whereas Aβ removal is largely associated with proteases and lysosomal enzymes. Subcellularlly, Aβ production together with Aβ precursor protein APP seems closely related with mitochondria, the major source of energy for the brain. Mitochondrial changes including increasing ROS production and reducing ATP generation are in an age-dependent manner. ROS-related oxidative stress induces more Aβ production, while Aβ and APP localized to mitochondrial membranes cause mitochondrial damage by elevating ROS production, blocking the transport of nuclear-encoded mitochondrial protein and disrupting ETC activities. However, the mechanisms of Aβ and APP transport into mitochondrial membranes are still unknown. Future work focus on this part might provide well understanding between mitochondria and APP as well as Aβ production, which might be helpful for exploring new compounds.

On the other hand, microglial cells play very important roles in the removal of accumulated Aβ not only by phagocytosis but also by releasing proteases such as IDE for degradation, and it is also associated with the innate immune system induced by the aggregated Aβ. Therefore, further researches are needed to find how to keep the clearance function of microglial cells without being impaired by the proinflammatory cytokines.

Although tremendous progress has been made in the development therapeutic strategies targeting Aβ, more work are still needed to find efficient drugs for curing AD. Network regulations of Aβ should be taken into consideration for the therapy approaches, and it would be instrumental to create good animal models and find more specific biomarkers for the Aβ-mediated pathogenesis of AD.

## Conflict of Interest Statement

The authors declare that the research was conducted in the absence of any commercial or financial relationships that could be construed as a potential conflict of interest.
